# Relationship Between Group 3 Innate Lymphoid Cells and Th17 in Human Nasopharynx-Associated Lymphoid Tissue and the Association With Pneumococcal Carriage

**DOI:** 10.1093/infdis/jiaf488

**Published:** 2025-10-17

**Authors:** Lualuaa Zaki, Rong Xu, Ravi Sharma, Madhan Krishnan, Samuel Leong, Stephen Derbyshire, Isobel Marks, Fabio Bagnoli, Paul S McNamara, Neil French, Qibo Zhang

**Affiliations:** Department of Clinical Infection, Microbiology and Immunology, Institute of Infection and Global Health, University of Liverpool, Liverpool; Department of Clinical Infection, Microbiology and Immunology, Institute of Infection and Global Health, University of Liverpool, Liverpool; ENT Department, Alder Hey Children's Hospital, Liverpool; ENT Department, Alder Hey Children's Hospital, Liverpool; ENT Department, Aintree University Hospital, Liverpool; ENT Department, Royal Surrey County Hospital, Guildford, United Kingdom; ENT Department, Royal Surrey County Hospital, Guildford, United Kingdom; Vaccines Research, GSK, Siena, Italy; Institute of Child Health, Alder Hey Children's Hospital, Liverpool; Department of Clinical Infection, Microbiology and Immunology, Institute of Infection and Global Health, University of Liverpool, Liverpool; Department of Clinical Infection, Microbiology and Immunology, Institute of Infection and Global Health, University of Liverpool, Liverpool; Department of Microbes, Infection and Immunity, School of Biosciences and Medicine, University of Surrey, Guildford, United Kingdom

**Keywords:** ILC3, nasopharynx-associated lymphoid tissue (NALT), pneumococcal carriage, *Streptococcus pneumoniae*, Th17

## Abstract

**Background:**

Nasopharyngeal carriage of *Streptococcus pneumoniae* (*Sp*) in children is common and considered a prerequisite of invasive disease. Group 3 innate lymphoid cells (ILC3) are considered critical in mucosal homeostasis. It is not known if ILC3 play a role in *Sp* carriage and how ILC3 interact with *Sp* in the nasopharynx.

**Methods:**

We characterized ILC3 in the nasopharynx-associated lymphoid tissue of children and adults and examined the relationship of ILC3 with Th17 cells and *Sp* carriage. We also analyzed the effects of *Sp* and *Staphylococcus aureus* on ILC3.

**Results:**

We show that the frequencies of natural cytotoxicity receptor (NCR)+ ILC3 and IL-22–producing ILC3 in the adenotonsillar cells of children were markedly higher than in adults. By contrast, the frequency of Th17 was considerably lower in children than adults. Interestingly, the frequency of NCR+ILC3 in children who were *Sp* carriage^+ve^ was higher than that of children who were *Sp* carriage^−ve^. Furthermore, stimulation of adenotonsillar cells with *Sp* culture supernatant induced a prominent ILC3 proliferative response and an increase in ILC3 number and level of HLA-DR expression, which correlated with a modest Th17 response. On the contrary, stimulation by *S aureus* culture supernatant reduced the ILC3 number and was accompanied by a strong Th17 response.

**Conclusions:**

The abundance of NCR+ILC3 in the nasopharynx of children is positively associated with *Sp* carriage. The positive relationship between ILC3 and *Sp* may suggest an important role of ILC3 in the nasopharyngeal carriage of *Sp*, and further investigation is warranted. Understanding local interactions between ILC3 and ILC3-promoting *Sp* or ILC3-repressing *S aureus* may lead to novel therapeutics against nasopharyngeal carriage and disease.

## Teaser

We demonstrated a positive relationship between nasopharynx-colonizing *Streptococcus pneumoniae* and group 3 innate lymphoid cells in the nasopharynx of children. Our findings suggest an important role for group 3 innate lymphoid cells in the nasopharyngeal carriage of *S pneumoniae* that presents a possible therapeutic target.

Bacterial carriage of the human nasopharynx is considered a prerequisite for invasive disease [1–3]. Among the common colonizers, *Streptococcus pneumoniae* (*Sp*) and *Staphylococcus aureus* (*Sa*) are important causes of invasive diseases in humans, although there appears to be an inverse relationship between the carriage status of *Sp* and *Sa*; that is, the 2 rarely co-colonize in the nasopharynx [3–5]. *Sp* carriage is common in young children, and their higher carriage rate gradually decreases with age into adulthood. It has been suggested that CD4+ T cells, and Th17 cells in particular, play a critical role in the nasopharyngeal clearance of *Sp* and the reduction of *Sp* carriage [6–8]. However, the cellular mechanisms that mediate and promote the carriage status of *Sp* during childhood remain unclear. Nasopharynx-associated lymphoid tissue (NALT), comprising adenoids and tonsils, is a major component of the mucosal immune system in the nasopharynx and a known induction site for immunity against upper respiratory pathogens [9]. Uniquely located in the nasopharynx and especially active during early childhood, NALT may play important roles in mediating local bacterial carriage and its clearance.

Current data from animal studies suggest that innate and adaptive immunity plays a role in mediating the homeostasis between the host and local microbiota in the gastrointestinal tract [10, 11]. In general, bacterial carriage in the nasopharynx is most common and intense during early childhood when adaptive immunity is still developing. Innate mechanisms may therefore play an important role in this period in mediating local homeostasis and bacterial carriage status in the nasopharynx.

Innate lymphoid cells are prevalent at mucosal sites and considered important in mucosal homeostasis and for the control of mucosal immunity [12]. Evidence from animal studies suggests that group 3 innate lymphoid cells (ILC3), specifically natural cytotoxicity receptor–positive ILC3 (NCR+ILC3), play a critical role in maintaining homeostasis with commensal carriage in the intestinal tract [10, 13, 14]. Recent studies also suggest an important role for intestinal ILC3 in regulating local CD4+ T-cell responses [11, 15].

In this study, we aimed to characterize the mucosal ILC3 in the NALT of children and adults and to elucidate the relationships among ILC3, Th17, and *Sp* carriage in the nasopharynx. We demonstrated an abundance of mucosal ILC3 in children, which is positively correlated with a higher *Sp* carriage rate in children. We also showed differential modulatory effects by *Sp* and *Sa* on ILC3, respectively promoting and repressing, which correlated with distinct Th17 responses.

## METHODS

### Patients and Samples

Adenotonsillar tissues were obtained from children (1.5–16 years, n = 38) and adults (17–40 years, n = 12) undergoing elective tonsillectomy due to upper airway obstruction at Alder Hey Children's Hospital and Aintree University Hospital in Liverpool and Royal Surrey County Hospital in Guildford, United Kingdom. Patients who had any known immunodeficiency were excluded from the study. Nasopharyngeal swabs were stored in STGG broth and kept at −80 °C before bacterial culture as described previously [8, 16]. The study was approved by the National Research Ethics Committee (14/SS/1058 and 22/NS/0092), and written consent was obtained in all cases from patients or their legal guardians.

### Cell Separation and Culture

Adenotonsillar mononuclear cells (MNCs) were isolated via density gradient centrifugation as described previously [17] and cultured at 4 × 10^6^ cells/mL in RPMI 1640 culture medium containing 10% heat-inactivated fetal bovine serum, HEPES, penicillin, and streptomycin (Thermo Fisher) in a 96-well plate [17]. In some experiments, CD45RO^+ve^ cells were depleted from the MNCs by magnetic cell sorting (Miltenyi Biotec) according to the manufacturer's instructions. CD45RO^+ve^ T-cell depletion removed memory T cells, including Th17, but retained naive T cells in the MNCs. The purity of the resulted CD45RO^−ve^ MNCs (>99%) was confirmed by flow cytometry, and the CD45RO^−^ MNCs were used for testing Th17 induction/differentiation.

### Characterization of ILC3 and Th17 and Intracellular Cytokine Staining

**Figure 1.**
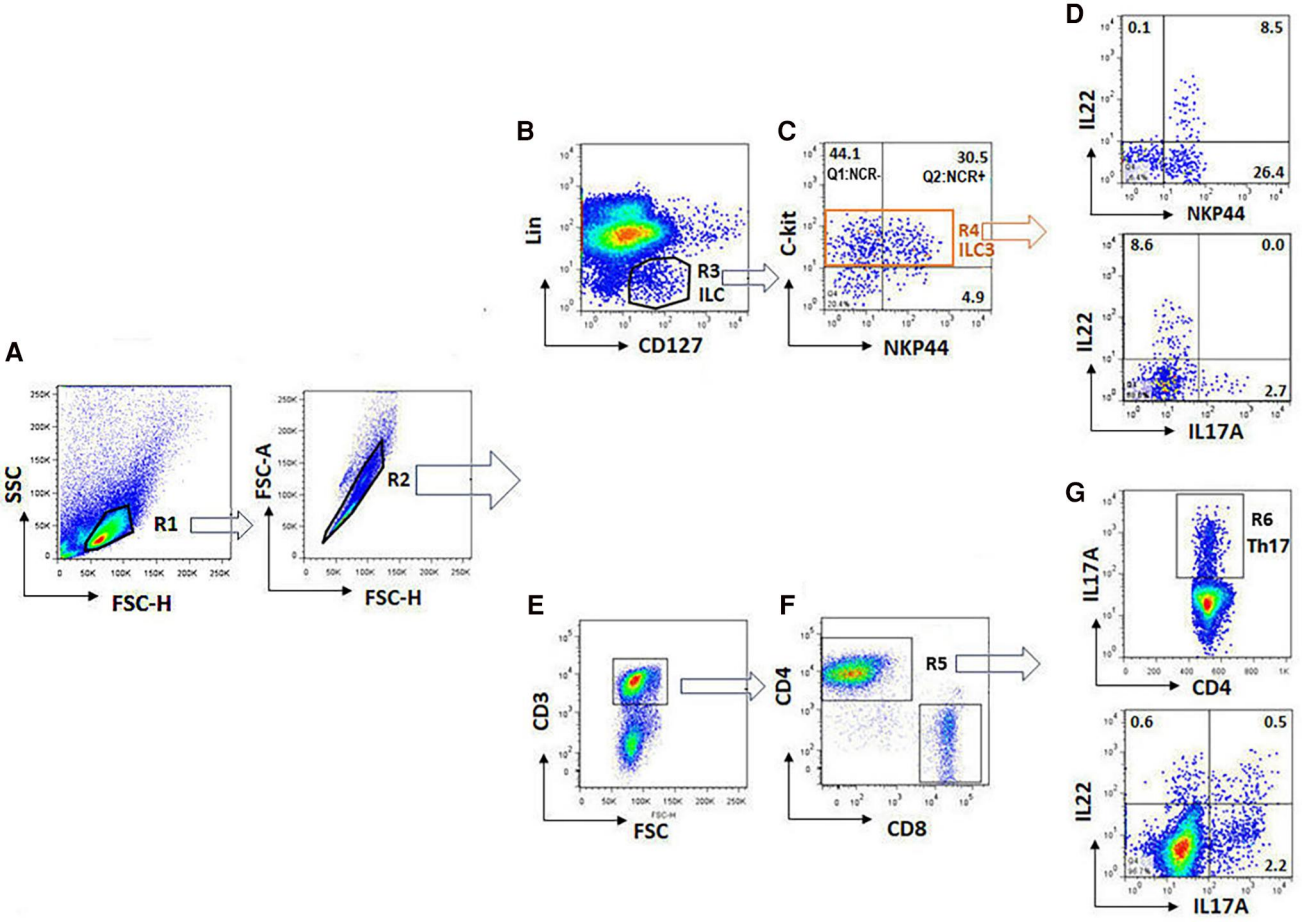
Flow cytometric gating strategy for characterization of group 3 innate lymphoid cells (ILC3) and Th17 in tonsillar mononuclear cells (MNCs). Tonsillar MNCs were cultured with and without PMA, ionomycin, and brefeldin A, followed by staining for surface markers and intracellular cytokines. *A*, Lymphocyte gating (R1) in MNCs by typical forward and sideward scatter, followed by singlet gating (R2), which were characterized for ILC3 or Th17. *B* and *C*, Lin–CD127+ innate lymphoid cells (R3) were gated into a c-kit+ ILC3 population (R4), which was divided into NKp44– (Q1, NCR–) and NKp44+ (Q2, NCR+) ILC3 subpopulations. *D*, ILC3 were further characterized for expressions of IL-22 and IL-17A, which were differentially produced by NKp44+ and NKp44– ILC3, respectively. *E–G*, CD3+/CD4+ T cells (R5) contain IL-17A–producing CD4+ T cells characterized as Th17 (R6) and IL-22–producing cells. One of 6 representative tonsillar tissue samples is shown. Abbreviation: NCR, natural cytotoxicity receptor.

For ILC3 identification, freshly isolated tonsillar MNCs were stained with fluorescence-labeled antibodies to surface markers, including lineage cocktail 1 (Lin-1; containing CD3/CD14/CD16/CD19/CD20/CD56), CD127, c-kit (CD117), and NKp44 (NCR; BioLegend), as followed by flow cytometry. ILC3 were defined as Lin-1^−^/CD127^+^/c-kit*^+^*, which were divided into NCR^+^ (NKp44^+ve^) and NCR^–^ (NKp44^−ve^) ILC3 (Figure 1*A*–*C*). IL-17A– and IL-22–producing ILC3 and Th17 were characterized by intracellular IL-17A and IL-22 staining (Figure 1*D*–*G*). Tonsillar MNCs were cultured at 4 × 10^6^/mL in a 96-well plate in RPMI 1640 culture medium supplemented with 10% fetal bovine serum and penicillin/streptomycin. After which, they were coincubated with PMA, ionomycin, and brefeldin A (eBiosciences) for 4 hours before cell harvesting, which was followed by cell surface staining (including CD3/CD4, Lin-1, CD127, CD117, and NKp44) and intracellular staining for IL-17A/IL-22 and RORγt based on a standard fixation/permeabilization procedure (eBiosciences) [18]. Flow cytometry was performed with BD FACS Celesta and data analyzed by FlowJo software.

### Preparation of *Sp* and *Sa* Culture Extracts and Cell Stimulation

Culture extracts of *Sp* and *Sa* were prepared from the culture supernatants of *Sp* and *Sa*. A standard encapsulated serotype 2 (D39) strain was used for *Sp* culture. For *Sa*, we included superantigenic strain FRI913 as SAg*+Sa* (positive for toxic shock syndrome toxin (tsst) and enterotoxins; BEI Resources/ATCC) and nonsuperantigenic strain USA300 LAC as SAg–*Sa* (negative for tsst and enterotoxins, kindly provided by Fabio Bagnoli [19]). Each strain was cultured in Todd Hewitt broth with 5% yeast extract to the exponential phase as described previously [18, 20]. After centrifugation, culture supernatant was concentrated 10-fold with Vivaspin concentrators (Vivascience/Sigma) as concentrated culture supernatant (CCS) of *Sp* and *Sa*. Protein concentrations of *Sp-*CCS and *Sa-*CCS were measured by a BCA protein assay (Fisher Scientific). *Sp-*CCS and *Sa-*CCS were then used (0.1–2 μg/mL) to stimulate tonsillar MNCs, followed by analysis of ILC3 and Th17 responses. Briefly, tonsillar MNCs were cultured at 4 × 10^6^/mL in a 96-well plate in RPMI 1640 culture medium and coincubated with *Sp-*CCS or *Sa-*CCS at predetermined concentrations for 3 days before cell harvesting. This was followed by cell surface staining (eg, CD4, Lin-1, CD127, CD117, NKp44, HLA-DR, and inducible costimulator [ICOS]) and by intracellular staining for IL-17A/IL-22 and RORγt following a standard fixation/permeabilization procedure (eBiosciences). The procedure for assessing cell proliferation via intracellular Ki67 staining of tonsillar MNCs after *Sp-*CCS stimulation for 3 days was performed following the manufacturer's instructions (BioLegend). The harvested cells were treated with cold 70% ethanol for 1 hour for permeabilization before being coincubated with BV421-labeled Ki67 antibody at room temperature in the dark for 30 minutes, followed by cell washing and resuspension and by flow cytometric analysis.

### Analysis of the Effect of RORgt Blockade on ILC3 and Th17 Responses

To determine the effect of RORgt blockade on an ILC3 or Th17 response, tonsillar MNCs were cultured at 4 × 10^6^ cells/mL in RPMI 1640 medium and coincubated for 2 hours with 0.5µM RORgt inhibitor (GSK805; Merck), which was reported to suppress a Th17 response and autoimmune condition [21], before being stimulated by SAg+*Sa-*CCS (0.1 μg/mL) for 18 hours. Cell culture supernatants were then removed for cytokine IL-17 analysis, and brefeldin A was added to the cell culture and incubated for a further 4 hours. The cells were then harvested and stained for ILC3 and Th17 surface markers, with intracellular cytokine staining for IL-17A, before flow cytometry with BD Celesta and data analysis with FlowJo software.

### Measurement of IL-17A Concentration

Culture supernatants of tonsillar MNCs with or without stimulation were measured for IL-17A concentrations by IL-17A enzyme-linked immunosorbent assay kits (eBioscence) following the manufacturer's instructions. Optical densities were read at 450 nm, and an IL-17A concentration (picograms per milliliter) for each sample was calculated from the IL-17A standard curve.

### Statistical Analysis

Comparisons between groups were analyzed with an unpaired *t* test with Welch correction, a paired Student *t* test, or analysis of variance. Statistical analyses were performed in Prism version 9 (GraphPad). *P* < .05 was considered statistically significant.

## RESULTS

### Characterization of ILC3 Reveals Distinct Relationship Between ILC3 and Th17 in Children and Adults

ILC3 and Th17 were characterized as detailed in the methods and shown in Figure 1. Tonsillar ILC3 (Lin-1^−^/CD127^+^/c-kit^+^, R4) were divided into NKp44^+ve^ (NCR+) and NKp44^−ve^ (NCR–) subpopulations (Q2 and Q1, respectively; Figure 1*C*). IL-22 expression in ILC3 was shown predominantly from NCR+ (NKp44^+ve^) and IL-17A mainly from NCR– (NKp44^−ve^). Th17 cells were identified as IL-17A–expressing CD4+ T cells (R6; Figure 1*G*).

**Figure 2.**
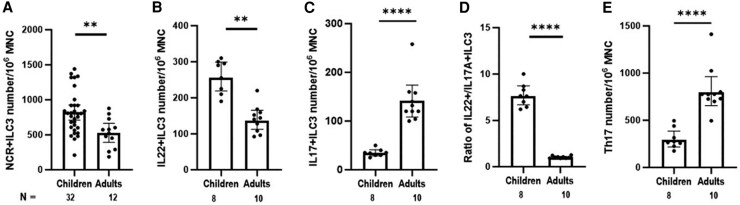
Frequencies of group 3 innate lymphoid cells (ILC3) in tonsillar mononuclear cells (MNCs) of children and adults. *A–C*, The frequencies of NCR+, IL-22–producing, and IL-17A–producing ILC3 in tonsillar MNCs were enumerated by flow cytometry and compared between children and adults. *D* and *E*, Comparison was also made in the ratio of IL-22+ILC3 to IL-17A+ILC3 and in Th17 frequency between children and adults. Data are presented as geometric mean and 95% CI. **P < .01. ****P < .0001. The number of patient samples analyzed for each comparison is indicated. Abbreviation: NCR, natural cytotoxicity receptor.

Higher frequencies of NCR+ILC3 (NKp44^+ve^), including IL-22^+ve^ ILC3 in tonsillar MNCs, were shown in children than adults (Figure 2*A* and 2*B*; *P* < .01). By contrast, IL-17^+ve^ ILC3 frequencies were lower in children than adults (Figure 2*C*; *P* < .01). Notably, the frequencies for IL-22^+ve^ ILC3 and IL-17A^+ve^ ILC3 in all adults were in general similar, with the mean ratio close to 1:1 (IL-22^+ve^ to IL-17A^+ve^ ILC3), whereas in children the mean ratio was 7.7:1—that is, the frequency of IL-22^+ve^ ILC3 was substantially higher than IL-17A^+ve^ ILC3 in children (Figure 2*D*; *P* < .001). Meanwhile, Th17 frequency in the tonsillar MNCs of children was markedly lower than in adults (Figure 2*E*; *P* < .0001), indicating a negative correlation between IL-22^+ve^ ILC3 and Th17 response in NALT.

### A Higher ILC3 Frequency in NALT Is Associated With *Sp* Carriage in Children

**Figure 3.**
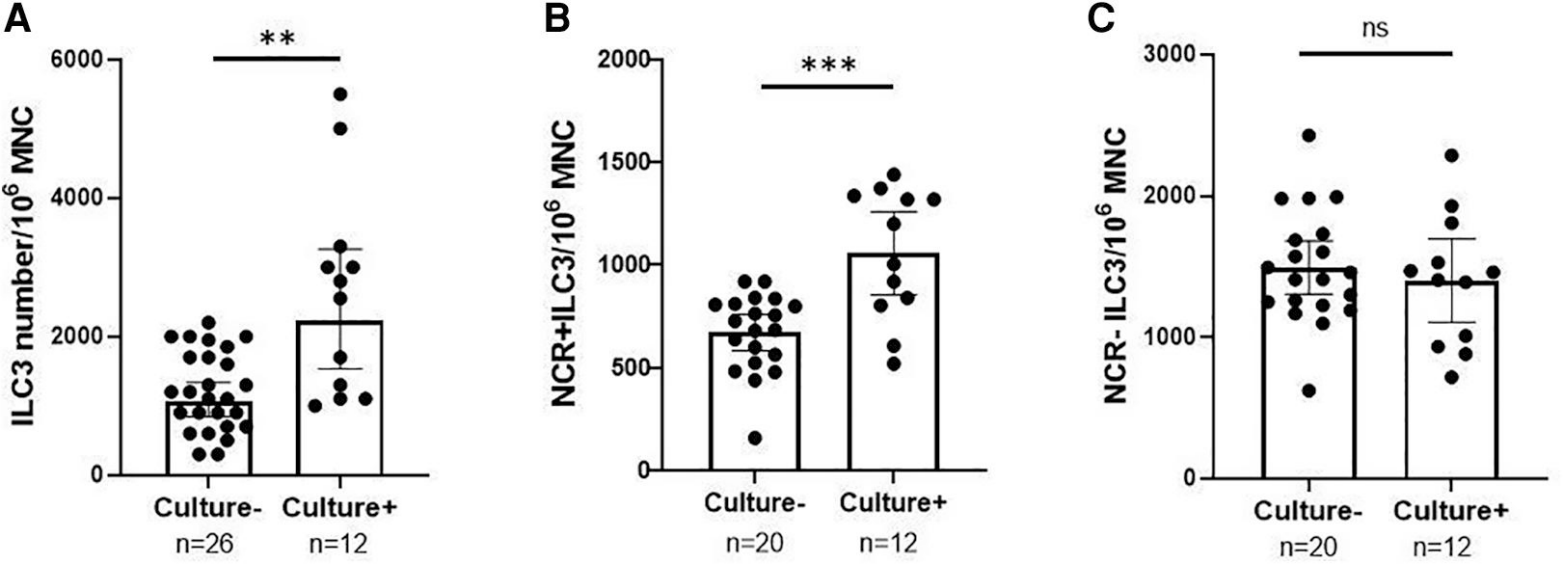
Relationship between the frequency of group 3 innate lymphoid cells (ILC3) in tonsillar mononuclear cells (MNCs) and *Sp* carriage in children. Geometric mean (95% CI) frequencies of ILC3 in tonsillar MNCs were compared between children who were *Sp* culture^+ve^ and *Sp* culture^−ve^ per the nasopharyngeal swabs: *A*, total ILC3; *B*, NCR+ILC3; *C*, NCR–ILC3. **P < .01. ***P < .001. Abbreviations: NCR, natural cytotoxicity receptor; ns, not significant; *Sp*, *Streptococcus pneumoniae*.

Previous studies suggested a critical role for Th17 in the clearance of *Sp* carriage [6–8]. Having shown a higher number of NCR+ and IL-22–producing ILC3 in children than in adults and an inverse association with Th17 frequency (Figure 2), we next examined whether this higher ILC3 frequency was associated with *Sp* carriage in children. Indeed, as shown in Figure 3*A*, the frequency of ILC3 in children who were *Sp* carriage^+ve^ was significantly higher than in children who were *Sp* carriage^−ve^. More interesting, this positive correlation was found only for the NKp44^+ve^ subset (Figure 3*B*; *P* < .01), not the NKp44^−ve^ ILC3 subset (Figure 3*C*). These results suggest that NCR+ (NKp44^+ve^) ILC3 may promote *Sp* carriage in the nasopharynx.

### *Sp* Induces ILC3 Proliferation and Increases ILC3 Frequency and HLA-DR Expression

**Figure 4.**
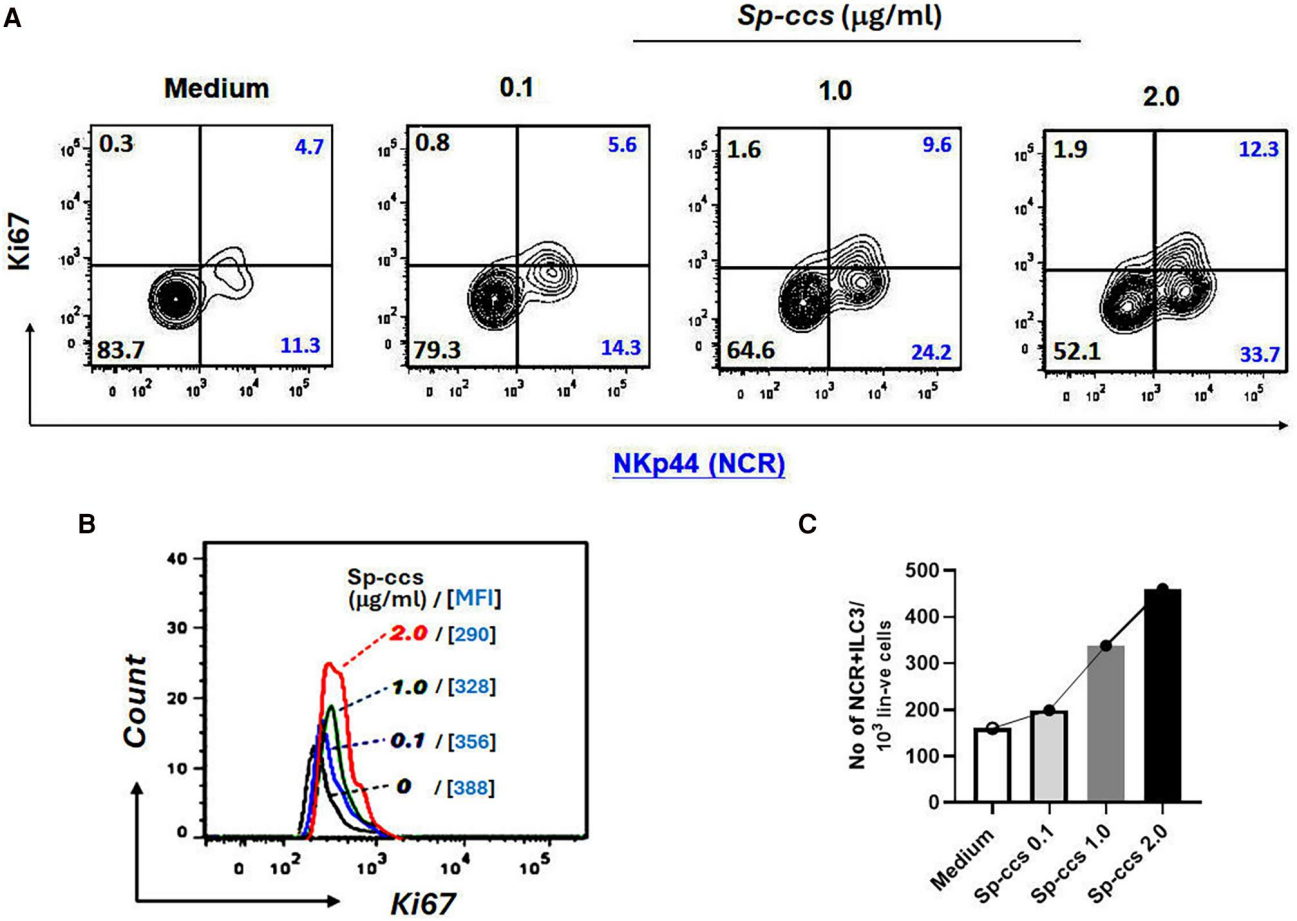
*Sp* culture supernatant (*Sp-*CCS) stimulates the proliferation of group 3 innate lymphoid cells (ILC3) in tonsillar mononuclear cells (MNCs). Tonsillar MNCs were stimulated with *Sp-*CCS at concentrations at 0.1, 1.0, and 2.0 μg/mL as compared with unstimulated control (medium only), followed by flow cytometric analysis for the NKp44(NCR)+ ILC3 proliferative response (Ki67^+ve^). Following *Sp-*CCS stimulation, a dose-dependent increase in Ki67^+ve^ NCR+ILC3 was shown in FACS contour plots (*A*; right upper quadrants as percentage of all Lin^−ve^ cells in tonsillar MNCs) and a FACS histogram (*B*; *Sp*-CCS concentrations and mean fluorescence intensity [MFI] values for each stimulation). *C*, Bar graph illustrates the increase in the total number of NCR+ILC3 (Ki67^+ve^ and Ki67^−ve^) among Lin^−ve^ cells in tonsillar MNCs following *Sp*-CCS stimulation. One of 4 representative patient samples is shown. Abbreviations: NCR, natural cytotoxicity receptor; *Sp*, *Streptococcus pneumoniae*.

To further examine the relationship between *Sp* and NCR+ILC3, we tested whether *Sp* could modulate the number of NCR+ILC3. Tonsillar MNCs were stimulated with a concentrated *Sp* culture supernatant (*Sp*-CCS), followed by ILC3 enumeration. *Sp*-CCS stimulation induced a remarkable proliferative (Ki67^+ve^) response in NCR+ (NKp44^+ve^) ILC3 (Figure 4*A*) and a dose-dependent increase in the total number of NCR+ILC3 (Ki67^+ve^ and Ki67^−ve^) among the Lin^−ve^ cells in tonsillar MNCs (Figure 4*B*).

**Figure 5.**
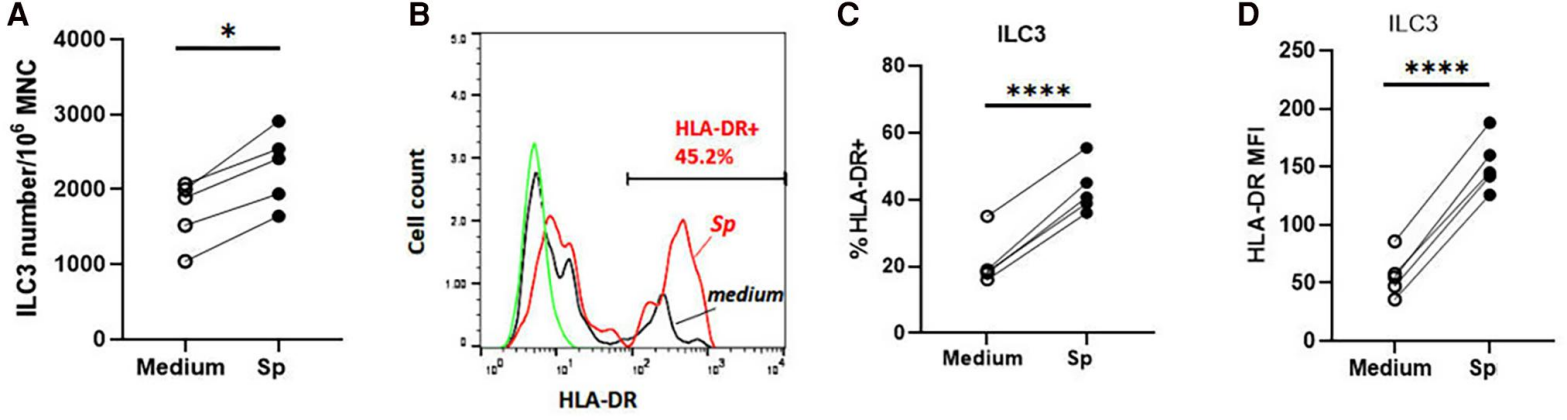
Effect of *Sp*-CCS on frequency of group 3 innate lymphoid cells (ILC3) and expression of HLA-DR. ILC3 frequency (*A*) and levels of HLA-DR expression on ILC3 (*B*–*D*) of tonsillar mononuclear cells (MNCs) are shown following *Sp-*CCS stimulation as compared with unstimulated MNC control (medium only). *B*, A representative FACS histogram illustrates tonsillar ILC3 HLA-DR expression following *Sp-*CCS stimulation: *Sp-*CCS (*Sp*, red), unstimulated control (medium, black), and isotype control (green). *C* and *D*, Percentage of HLA-DR+ILC3 and mean fluorescence intensity of HLA-DR expression following *Sp-*CCS stimulation as compared with medium control. *P < .05. ****P < .0001. n = 5. Abbreviations: CCS, culture supernatant; *Sp*, *Streptococcus pneumoniae*.

*Sp*-CCS stimulation was shown to increase the frequency of total ILC3 (Lin–/CD127+/c-kit+) in tonsillar MNCs (Figure 5*A*) and the frequency HLA-DR+ILC3, as well as the level of HLA-DR expression (mean fluorescence intensity) of ILC3, as compared with unstimulated controls (Figure 5*B*–5*D*; *P* < .0001). In addition, we showed that *Sp-*CCS stimulation markedly upregulated ICOS expression in ILC3 and CD4+ T-cell populations (Supplementary Figure 1).

### *Sa* Represses ILC3 but Activates a Strong Th17 Response

**Figure 6.**
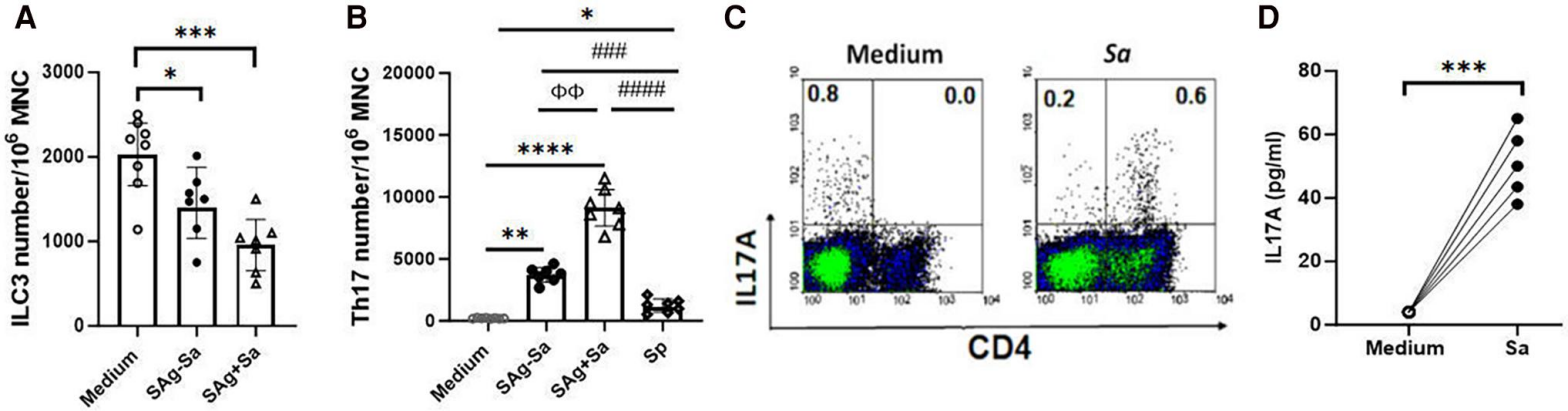
Effect of *Sa-*CCS stimulation on group 3 innate lymphoid cells (ILC3) and Th17 response. *A* and *B*, Tonsillar mononuclear cells (MNCs) were stimulated by superantigenic *Sa*-CCS (SAg+*Sa*) or nonsuperantigenic *Sa-*CCS (SAg–*Sa*), followed by enumerating ILC3 and Th17 as compared with unstimulated MNC control (medium) or *Sp-*CCS stimulated MNCs. Compared with medium control: *P < .05. ***P < .001. ****P < .0001. SAg+*Sa* vs SAg–*Sa*: ^ΦΦ^*P* < .01. Compared with *Sp*-stimulated MNCs (n = 7): ^###^*P* < .001. ^####^*P* < .0001. Data are presented as geometric mean and 95% CI. *C* and *D*, A representative FACS dot plot demonstrates induction of CD4+Th17 cells in CD45RO^−ve^ MNCs and IL-17A concentrations in CD45RO^−ve^ MNCs following SAg+*Sa*-CCS stimulation as compared with unstimulated cell controls (medium only). ***P < .001 (n = 5). Abbreviations: CCS, culture supernatant; *Sa*, *Staphylococcus aureus*; *Sp*, *Streptococcus pneumoniae*.

We next examined whether *Sa*, another common nasopharyngeal colonizer, has a similar effect as *Sp* on ILC3. Surprisingly, stimulation of tonsillar MNCs by *Sa-*CCS elicited a marked decrease in ILC3 frequency as compared with unstimulated MNCs (Figure 6). This effect was not due to any toxic or cell-killing effect by *Sa*-CCS, as total cell viability was not reduced as compared with unstimulated MNCs (data not shown). While superantigen-expressing (SAg^+ve^) and non–superantigen-expressing (SAg^−ve^) *Sa*-CCS elicited a decrease in ILC3 as compared with unstimulated MNCs, a more prominent reduction in ILC3 was shown in tonsillar MNCs stimulated by SAg^+ve^ than by SAg^−ve^  *Sa*-CCS (Figure 6*A*; *P* < .01). Conversely, a stronger Th17 response was elicited by SAg^+ve^ than by SAg^−ve^  *Sa-*CCS (Figure 6*B*), and SAg^+ve^ and SAg^−ve^  *Sa-*CCS were shown to activate a markedly stronger Th17 response than *Sp-*CCS.

We further examined whether the reduction in ILC3 by *Sa-*CCS bore any relationship with Th17 induction using Th17-depleted (CD45RO^−ve^) tonsillar MNCs. Stimulation of CD45RO^−ve^ MNCs with *Sa*-CCS for 7 days resulted in newly induced IL-17A–producing CD4+ T cells (Th17), as shown in Figure 6*C*. *Sa-*CCS stimulation reduced the number of IL-17A+ILC3, as shown in the CD4– population, which was accompanied by the detection of IL-17A–producing CD4+ T cells (ie, Th17). This induction of Th17 was supported by the detection of IL-17A in CD45RO^−ve^ MNC culture following *Sa-*CCS stimulation (Figure 6*D*).

### Effect of RoRγt Blockade on ILC3 and Th17 Responses

**Figure 7.**
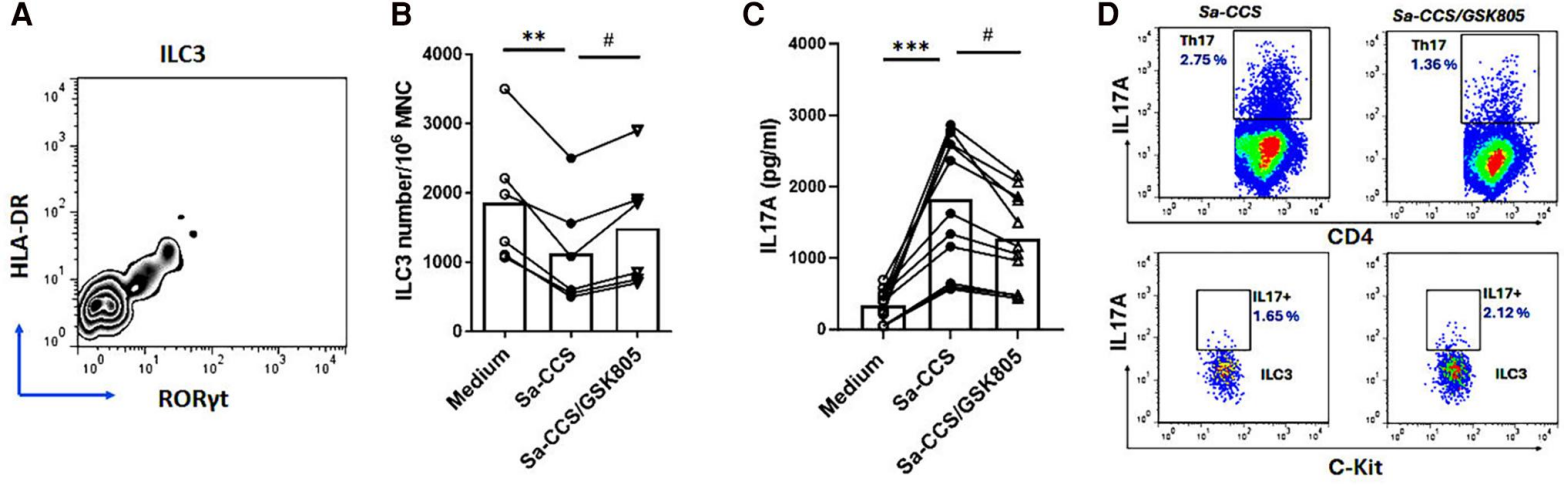
Effect of RORγt blockade on group 3 innate lymphoid cells (ILC3) and Th17 responses in tonsillar mononuclear cells (MNCs). *A*, A representative FACS zebra plot of ILC3 cells in tonsillar MNCs demonstrates coexpression of RORγt and HLA-DR (1 of 4 patient samples is shown). *B* and *C*, Effect of RORγt inhibitor (GSK805) on SAg*+Sa-*CCS elicited a decrease in ILC3 frequency (n = 6), and that on SAg*+Sa-*CCS activated an IL-17A response in tonsillar MNCs (n = 10). Compared with unstimulated control (medium only): ^**^P < .01. ***P < .001. Compared with SAg*+Sa-*CCS following GSK805 treatment: ^#^*P* < .01. *D*, Intracellular cytokine staining FACS dot plots demonstrate that the *Sa-*CCS–activated Th17 (CD4+IL-17A+) response was reduced, whereas IL-17A+ILC3 frequency was modestly increased by GSK805 treatment (1 of 4 representative samples is shown). Abbreviations: CCS, culture supernatant; *Sa*, *Staphylococcus aureus*.

RoR*γ*t is critical for Th17 activation and expressed by ILC3 [10]. There was a subset of tonsillar ILC3 expressing RoR*γ*t, which was concordant with the HLA-DR–expressing ILC3 subset (Figure 7*A*). To examine whether the *Sa*-elicited decrease of ILC3 and/or activation of a Th17 response in tonsillar MNCs involved RoR*γ*t, GSK805 (a ROR*γ*t inhibitor) was used to blockade RoR*γ*t signaling before cell stimulation by *Sa-*CCS. RoR*γ*t blockade partly reversed the effect of *Sa-*CCS on ILC3 reduction (Figure 7*B*) and in the meantime reduced the *Sa-*CCS–activated Th17 response in tonsillar MNCs (Figure 7*C*).

## DISCUSSION

Our results demonstrate an abundance of NCR+ and IL-22–producing ILC3 in the nasopharynx of children. In addition, a higher NCR+ILC3 frequency in the NALT is associated with the nasopharyngeal carriage of *Sp*.

NCR+ILC3 is a major subset of ILC3, which are known to express IL-22 and play a critical role in mucosal homeostasis in the intestine [10, 13]. We showed here that tonsillar NCR+ILC3 express predominantly IL-22 (Figure 1) and that children had a remarkably higher frequency of IL-22–producing ILC3 than IL-17A–producing ILC3 at a ratio of 7.7:1.0, as compared with 1:1 in adults (Figure 2). This high abundance of IL-22–producing ILC3 in children correlated with markedly lower Th17 frequency relative to adults. This interesting inverse relationship between ILC3 and Th17 in the NALT raises the possibility that IL-22–producing NCR+ILC3 may regulate a Th17 response in the nasopharynx and modulate *Sp* carriage status.

Considering the importance of Th17 in the clearance of bacterial carriage and the role of ILC3 in inhibiting Th17 differentiation [22–24], abundant ILC3 in the nasopharynx would potentially downregulate Th17 and favor *Sp* carriage. Indeed, we demonstrated that a higher ILC3 frequency in NALT was associated with *Sp* carriage in children and that NCR+ILC3, not NCR–ILC3, were positively associated with *Sp* carriage (Figure 3). Furthermore, *Sp-*CCS stimulation induced NCR+ILC3 proliferation and increased ILC3 frequency in tonsillar MNCs. These findings suggest an interesting reciprocal relationship and interaction between NCR+ILC3 and *Sp*—specifically, *Sp* colonization promotes NCR+ILC3 and vice versa. A higher number of NCR+ILC3 may provide enhanced regulation of a Th17 response and help establish and maintain stable *Sp* carriage.

The molecular mechanisms by which ILC3 promote *Sp* carriage are as yet unclear, although it may involve the actions of MHC-II+ILC3 and IL-22–producing ILC3. MHC-II+ILC3 were shown to inhibit a CD4+ T-cell response, including Th17, through antigen presentation [15], and we show here that *Sp* stimulated an increase in the numbers of HLA-DR+ILC3 and the levels of HLA-DR expression. Furthermore, intestinal commen­sals could modulate IL-22–producing innate lymphoid cells and drive IL-22 production in ILC3 [25, 26]. *Sp* may act similarly as a commensal in the nasopharynx and drive IL-22–producing ILC3 proliferation. A previous study in a mouse model demonstrated the increase in IL-22+ILC3 in the lung during *Sp* infection [27]. We previously showed that *Sp* stimulation induced the release of an array of cytokines [17, 28] from tonsillar cells, which may promote ILC3 proliferation, as ILC3 constitutively express cytokine receptors known to promote ILC3 [29]. Although IL-22 and HLA-DR do not appear to coexpress in ILC3 [30], they may act synergistically to help maintain *Sp* carriage.

Furthermore, *Sp-*CCS stimulation was shown to markedly upregulate the expression of ICOS expression in the ILC3 and CD4+ T-cell population (Supplementary Figure 1). It has been shown that ICOS signaling drives ILC3 proliferation [31]. It is therefore likely that *Sp* contains property that activates the ICOS signaling pathway, which promotes ILC3 proliferation. It is worth noting that the effects of *Sp-*CCS on upregulating ICOS expression and promoting ILC3 proliferation could be markedly reduced by heat treatment (thus heat labile; data not shown), suggesting that this activating property in the *Sp* culture supernatant is of a protein nature.

It is interesting that *Sp*-CCS induced an increase in ILC3 frequency, which correlated with only a modest Th17 response, whereas *Sa-*CCS suppressed ILC3 frequency, which correlated with a strong Th17 response. Also, the extent of the suppression of ILC3 frequency by the superantigenic and nonsuperantigenic *Sa* strains positively correlated with the magnitude of the Th17 response. Furthermore, experiments on Th17 induction demonstrated that *Sa*-CCS's suppression on ILC3 correlated with the development of newly differentiated Th17 (Figure 6). These findings are concordant with the hypothesis that ILC3 contribute to the regulation of Th17 and with reports that a reduction in ILC3 was accompanied by the increase in Th17 in patients with colonic inflammation [15]. The suppression of ILC3 by *Sa* may lead to reduced control of Th17 and contribute to a strong Th17 response.

Our findings suggest distinct differences and interactions between *Sp*- and *Sa*-induced ILC3 and Th17 responses and their effect on *Sp* carriage. Children are susceptible to *Sp* colonization, which promotes NCR+ILC3 frequency and function, leading to a controlled Th17 response and a stable *Sp* carriage status. When *Sa* colonizes, it suppresses ILC3, leading to an enhanced Th17 response that promotes the clearance of *Sp*. The fact that *Sa* rarely co-colonizes the nasopharynx with *Sp* is consistent with this hypothesis [5].

The strong Th17 response activated by *Sa* has the potential to cause tissue inflammation, which under normal conditions is likely controlled by regulatory mechanisms, including ILC3 in addition to T regulatory cells [8, 18]. However, carriage of superantigenic *Sa* has been associated with some autoimmune conditions [32], and strategies against these inflammatory conditions have been explored, including blockade of the Th17 induction pathway. RORγt is critical for Th17 activation and is also expressed in ILC3. We showed that RORγt signaling blockade significantly inhibited an *Sa*-activated Th17 response with no inhibition on ILC3. Instead, prior RORγt blockade ameliorated an *Sa-*induced decrease in ILC3, including IL-17A–producing ILC3. This supports that an *Sa*-induced strong Th17 response could be regulated by inhibiting RORγt transcription without affecting the ILC3 regulatory effect [33].

In conclusion, the abundant number of NCR+ILC3 in NALT is associated with the nasopharyngeal carriage of *Sp* in children. The positive relationship between *Sp* and ILC3 may suggest an important role of ILC3 in the nasopharyngeal carriage of *Sp*, and further investigation is warranted. *Sp* induces a NCR+ILC3 proliferative response that may promote a regulated/reduced Th17 response and help establish and maintain *Sp* carriage. The differential modulatory effects by *Sp* and *Sa* on ILC3, respectively promoting and repressing, are associated with distinct Th17 responses. Further studies are planed to investigate the molecular mechanisms on how ILC3 interact with local colonizers, including *Sp* and *Sa*, thereby identifying an effective strategy to control bacterial carriage and prevent disease.

## Supplementary Material

jiaf488_Supplementary_Data

## Supplementary Data

Supplementary materials are available at *The Journal of Infectious Diseases* online (http://jid.oxfordjournals.org/). Supplementary materials consist of data provided by the author that are published to benefit the reader. The posted materials are not copyedited. The contents of all supplementary data are the sole responsibility of the authors. Questions or messages regarding errors should be addressed to the author.
