# Supplementary material for: Relationship Between Group 3 Innate Lymphoid Cells and Th17 in Human Nasopharynx-Associated Lymphoid Tissue and the Association With Pneumococcal Carriage
Source: J Infect Dis. 2025 Oct 17;232(6):e972–80. doi: 10.1093/infdis/jiaf488 (PMC12718059; doi:10.1093/infdis/jiaf488)
Supplement: jiaf488_Supplementary_Data [file jiaf488_supplementary_data.zip › Supplementary Figure 1 legends.docx]

**Supplementary Data**

***Supplementary figure 1.*** **Effect of *Sp* culture supernatant (*Sp-CCS)* stimulation on expressions of ICOS in tonsillar MNC.** Tonsillar MNC were cultured in RPMI1640 medium (4x10^6^ cells/ml) and stimulated with *Sp-CCS* (1.0 μg/ml) for 3 days as compared with unstimulated (medium only) control, followed by flowcytometric analysis for ICOS expression in tonsillar MNC including C-kit+ ILC3 and CD4+ T cells. Marked increases in ICOS expression were shown in both C-kit+ILC3 and CD4+ T cells in tonsillar MNC following Sp-CCS stimulation. One of 4 representative patient samples is shown.
