# Supplementary figures and images for: Relationship Between Group 3 Innate Lymphoid Cells and Th17 in Human Nasopharynx-Associated Lymphoid Tissue and the Association With Pneumococcal Carriage
Source: J Infect Dis. 2025 Oct 17;232(6):e972–80. doi: 10.1093/infdis/jiaf488 (PMC12718059; doi:10.1093/infdis/jiaf488)

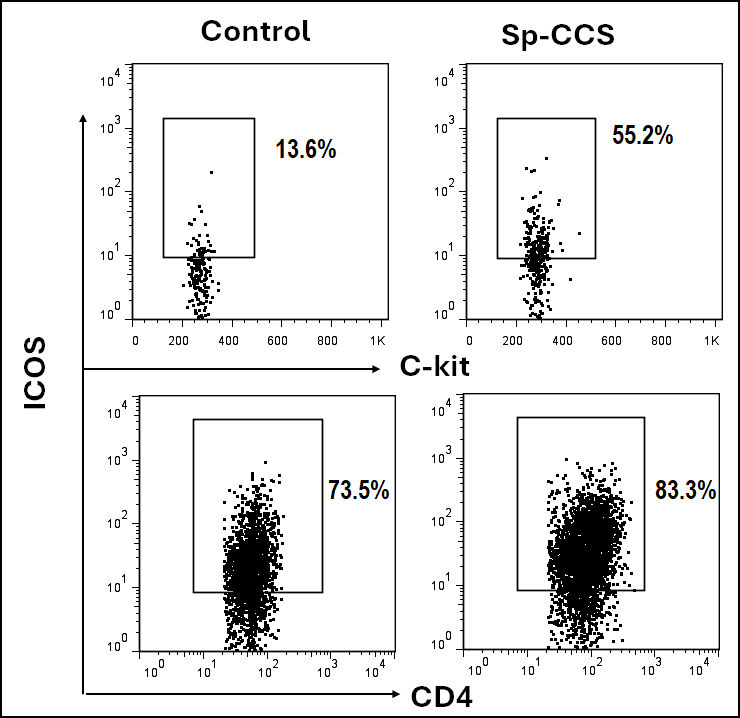

Supplement: jiaf488_Supplementary_Data [file jiaf488_supplementary_data.zip › Supplementary Figure 1.jpg]
